# Comparative Analysis of Irrigation Mist and CO_2_ vs. Direct CO_2_ Blower in On-Pump Coronary Artery Bypass Grafting Anastomosis: Efficacy, Efficiency, and Fibrillation upon De-Clamping and Micro-Embolic Gas Activity Incidence

**DOI:** 10.3390/medicina60122035

**Published:** 2024-12-10

**Authors:** Ignazio Condello, Giuseppe Speziale, Flavio Fiore, Giuseppe Nasso

**Affiliations:** Department of Cardiac Surgery, Anthea Hospital GVM Care and Research, Via Camillo Rosalba 35/37, 70124 Bari, Italy

**Keywords:** coronary artery bypass grafting (CABG), ClearView™ Blower/Mister, CO_2_ blower, micro-embolic gas activity, transesophageal echocardiography (TEE), aortic de-clamping, intraoperative visualization, fibrillation, myocardial revascularization, tissue desiccation, surgical outcomes

## Abstract

*Background and Objectives:* In coronary artery bypass grafting (CABG) on pump, achieving optimal visualization is critical for surgical precision and safety. The use of blowers to clear the CABG anastomosis poses risks, including the formation of micro-embolic gas bubbles, which can be insidious and increase the risk of cerebral or myocardial complications. This retrospective study compares the effectiveness of the use of irrigation mist and CO_2_ versus a direct CO_2_ blower without irrigation in terms of visualization, postoperative fibrillation, and micro-embolic gas activity. *Materials and Methods:* The study involved 40 patients who underwent on-pump CABG, with 20 patients assigned to the irrigation mist and CO_2_ group (ClearView™) and 20 to the direct CO_2_ blower group. Primary outcomes included the quality of intraoperative visualization, the incidence of fibrillation at aortic de-clamping, and the presence of micro-embolic gas activity detected via transesophageal echocardiography (TEE) in the cardiac chambers. *Results:* Patients in the irrigation mist and CO_2_ group experienced superior visualization and reduced tissue desiccation. Fibrillation at the time of aortic de-clamping occurred in two patients (10%) using the irrigation mist and CO_2_, compared to eight patients (40%) using the direct CO_2_ blower. Additionally, TEE monitoring revealed lower levels of micro-embolic gas activity in the irrigation mist and CO_2_ group, indicating a potential reduction in gas embolization risk. *Conclusions:* The irrigation mist and CO_2_ system not only provides enhanced visualization during CABG but also significantly reduces the incidence of fibrillation during aortic de-clamping and micro-embolic gas activity. These findings suggest improved patient safety and outcomes, highlighting the irrigation mist and CO_2_ system as a potentially safer alternative to direct CO_2_ blowing in the context of myocardial revascularization.

## 1. Introduction

Coronary artery bypass grafting (CABG) remains a cornerstone in the surgical treatment of advanced coronary artery disease, offering long-term survival benefits and symptom relief for patients with multivessel coronary artery blockages. In on-pump CABG, where cardiopulmonary bypass is employed, ensuring a clear and unobstructed view of the surgical site is critical for precise anastomosis and optimal graft placement [1,2]. However, achieving and maintaining adequate visualization can be challenging due to continuous bleeding and the potential desiccation of tissues caused by commonly used CO_2_ blowers. Traditionally, CO_2_ blowers have been utilized to clear blood from the surgical field, but direct CO_2_ blowing can lead to tissue dehydration, fragility, and impaired healing. Moreover, this technique has been associated with the formation of micro-embolic gas bubbles, which pose significant risks of neurological and myocardial complications [3]. These micro-emboli, although not always immediately detectable, may cause cerebral or coronary ischemia and contribute to post-surgical morbidity. The irrigation mist and CO_2_ system was originally designed for off-pump coronary artery surgery, providing a dual-action approach that combines CO_2_ blowing with a gentle irrigation mist. This system aims to enhance intraoperative visualization while preserving tissue hydration and integrity [4]. The irrigation mist helps maintain moisture on the delicate myocardial and vascular tissues, reducing the risk of desiccation and minimizing potential tissue damage. However, its use in on-pump CABG has not been widely studied, raising questions about its effectiveness and safety in this particular surgical context. In this retrospective comparative analysis, we sought to evaluate the efficacy of irrigation mist and CO_2_ in on-pump CABG, comparing it with the traditional direct CO_2_ blower [5]. We focused on key clinical outcomes, such as intraoperative visualization, the incidence of fibrillation during aortic de-clamping, and the presence of micro-embolic gas activity detected via transesophageal echocardiography (TEE) within the cardiac chambers. The goal of this study is to determine whether the irrigation mist and CO_2_ system, initially developed for off-pump procedures, offers distinct advantages when applied to on-pump CABG, potentially improving surgical performance and patient safety.

## 2. Materials and Methods

**Figure 1:**
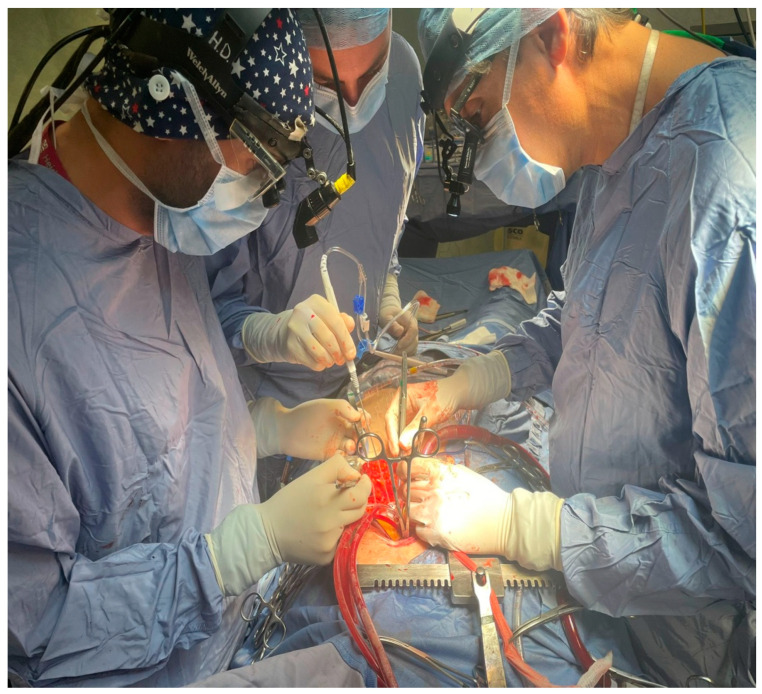
Perioperative use of the irrigation mist and CO_2_ during CABG.

**Figure 2:**
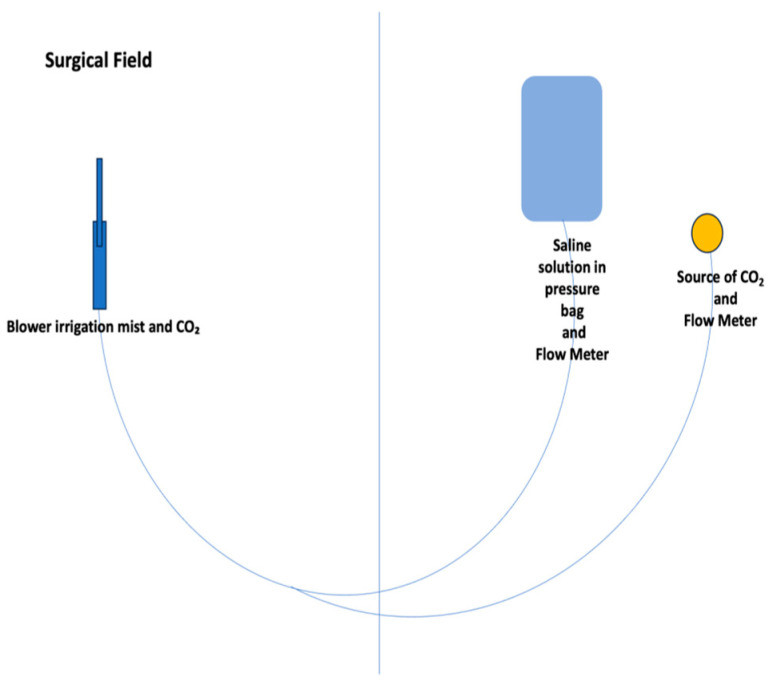
Irrigation mist and CO_2_ sketch.

**Figure 3:**
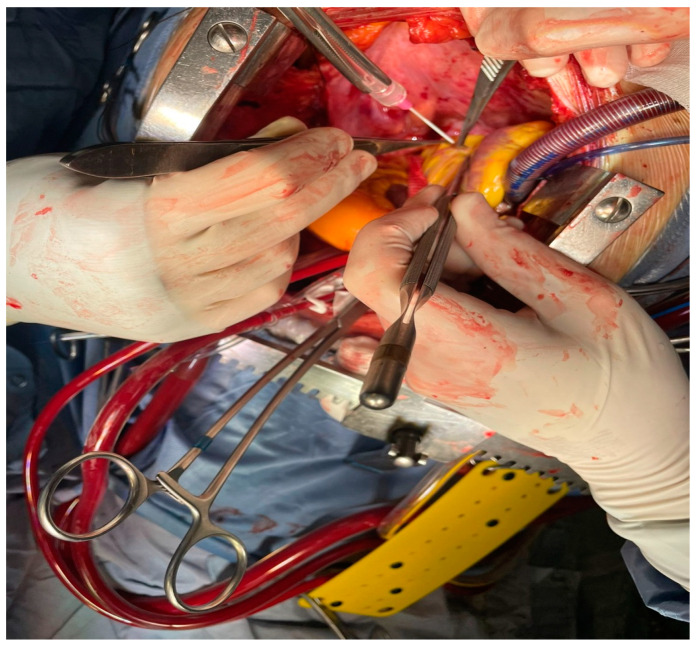
Perioperative use of the traditional direct CO_2_ blower.

This retrospective study compares two historical periods of on-pump coronary artery bypass grafting (CABG) at our institution, focusing on the use of the irrigation mist and CO_2_ (ClearView™ Blower/Mister by Medtronic, Minneapolis, MN, USA) (Figure 1 and Figure 2) versus the traditional direct CO_2_ blower (Figure 3). The aim was to assess the impact of these two methods on intraoperative visualization, the incidence of fibrillation during aortic de-clamping, and the presence of micro-embolic gas activity detected via transesophageal echocardiography (TEE).

*Study Design and Population*

The study included 40 patients who underwent elective on-pump CABG, divided into the following two groups:

- Group 1 (n = 20): patients operated on during a period before the introduction of the irrigation mist and CO_2_, where direct CO_2_ blowers were used;
- Group 2 (n = 20): patients operated on after the ClearView™ Blower/Mister system became available and was routinely used during surgery.

*Intervention*

- Group 1 (Direct CO_2_ Blower): Patients in this group underwent CABG using a traditional direct CO_2_ blower to clear blood from the surgical field. No irrigation mist was applied, and the CO_2_ blower was operated continuously during critical stages of the procedure, including the anastomosis phase;
- Group 2 (Irrigation Mist and CO_2_): patients in this group received CABG using the irrigation mist and CO_2_, which combines a CO_2_ blower with a fine irrigation mist to improve visualization and maintain tissue hydration during surgery.

*Outcomes Assessed*

- Visualization Quality: assessed subjectively by the operating surgeon, rated on a scale from 1 (poor) to 5 (excellent), and recorded in operative notes;
- Incidence of Fibrillation During Aortic Declamping: the number of patients experiencing fibrillation immediately after aortic cross-clamp removal was documented;
- Microembolic Gas Activity: transesophageal echocardiography (TEE) was used intraoperatively to detect the presence of microembolic gas bubbles in the cardiac chambers during and after aortic declamping.

*Cardioplegia delivery*

In both groups, a normothermic blood cardioplegia protocol was employed using a Saint Thomas solution, administered at regular intervals of every 20 min. This approach was chosen to provide consistent myocardial protection throughout the procedure, maintaining cardiac tissue viability during periods of ischemia. The cardioplegia solution was delivered at normothermic temperature to balance myocardial protection with metabolic demands, aiming to minimize ischemic damage during aortic cross-clamping.

*Statistical Analysis*

A comparative statistical analysis was conducted between the two groups. Continuous variables, such as the number of anastomoses and visualization quality, were compared using the Student’s *t*-test, while categorical variables like fibrillation incidence were analyzed using the chi-square test. A *p*-value of <0.05 was considered statistically significant. This comparative analysis allowed for an evaluation of the irrigation mist and CO_2_ system’s effectiveness in on-pump CABG compared to traditional methods, focusing on key outcomes including tissue preservation, fibrillation risk, and micro-embolic gas activity.

## 3. Results

### Patient and Surgical Characteristics

The demographic and baseline clinical characteristics of the two groups were comparable, with no significant differences in age, gender distribution, or preoperative risk factors, such as left ventricular ejection fraction (LVEF), diabetes, hypertension, or hyperlipidemia. Additionally, the surgical characteristics, including the number of proximal and distal anastomoses, and the use of specific grafting techniques, were similar between the two groups (Table 1).

- Proximal Anastomoses: the average number of proximal anastomoses was 2.3 ± 0.6 in Group 1 (direct CO_2_ blower) and 2.4 ± 0.5 in Group 2 (the irrigation mist and CO_2_);
- Distal Anastomoses: the average number of distal anastomoses was 3.1 ± 0.8 in Group 1 and 3.2 ± 0.7 in Group 2;
- Grafting Techniques: use of the Y-mammary graft was 40% in Group 1 and 50% in Group 2, while sequential grafting with the mammary artery and saphenous vein was performed in 60% of Group 1 and 50% of Group 2 patients.

*Visualization Quality*

**Figure 4:**
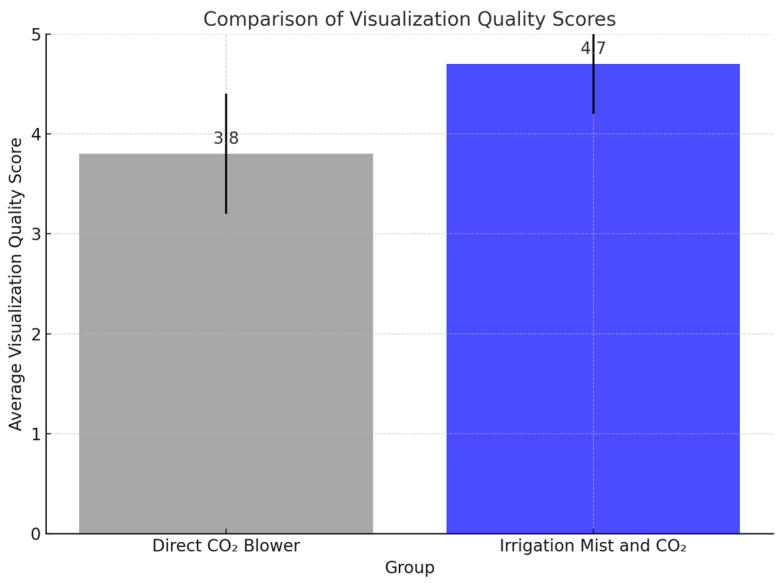
Bar graph comparing the visualization quality scores between the two groups.

The quality of visualization, as subjectively assessed by the three operating surgeon, was significantly better in Group 2, where the irrigation mist and CO_2_ was used. The average visualization score was 4.7 ± 0.5 in Group 2, compared to 3.8 ± 0.6 in Group 1 (*p* < 0.01). Surgeons in Group 2 consistently reported clearer visual fields with reduced blood obscuration and less tissue desiccation compared to Group 1 (Figure 4).

*Incidence of Fibrillation During Aortic De-Clamping*

A marked difference in the incidence of fibrillation during aortic de-clamping was observed between the two groups. In Group 1 (direct CO_2_ blower), eight patients (40%) experienced fibrillation at the time of de-clamping, whereas in Group 2 (irrigation mist and CO_2_), only two patients (10%) exhibited fibrillation (*p* = 0.03). This significant reduction suggests that the irrigation mist and CO_2_ system may help to stabilize the myocardium during this critical phase of surgery, potentially due to better tissue hydration, reduced stress on the cardiac tissues, and reduction of gaseous micro-emboli delivery.

*Micro-Embolic Gas Activity*

Transesophageal echocardiography (TEE) revealed a significantly lower incidence of micro-embolic gas activity in the cardiac chambers in Group 2 compared to Group 1. Micro-embolic bubbles were detected in 10 patients (50%) in Group 1, while only 3 patients (15%) in Group 2 showed evidence of micro-embolic gas activity (*p* = 0.02). The reduced micro-embolic gas activity in the irrigation mist and CO_2_ group suggests that the irrigation mist component may help mitigate gas embolization, potentially reducing the risk of neurological and myocardial complications postoperatively.

*Surgical Efficiency*

Although both groups underwent similar surgical procedures, a trend toward shorter total operative times was observed in Group 2. The improved visualization afforded by the ClearView™ system contributed to more efficient anastomosis construction, with Group 2 surgeries being approximately 10–15 min shorter on average compared to Group 1. However, this difference did not reach statistical significance (*p* = 0.07).

*Postoperative Outcomes*

**Figure 5:**
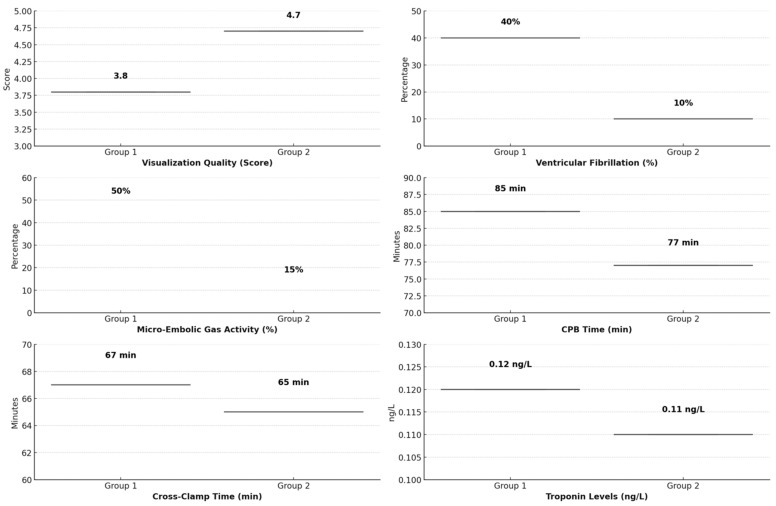
Comparative analysis of key perioperative outcomes in Group 1 (direct CO_2_ blower) vs. Group 2 (irrigation mist and CO_2_).

No significant differences were observed in major postoperative complications, such as graft failure, infection, or prolonged ICU stays, between the two groups. Both groups had similar rates of recovery and postoperative hospital stay duration, indicating that while intraoperative conditions improved with the irrigation mist and CO_2_ system, the overall patient recovery was comparable between the groups (Table 2) (Figure 5).

## 4. Discussion

This retrospective comparative study evaluated the use of an irrigation mist and CO_2_ system versus the traditional direct CO_2_ blower in on-pump coronary artery bypass grafting (CABG). By focusing on key outcomes such as intraoperative visualization, ventricular fibrillation during aortic de-clamping, and micro-embolic gas activity, the study highlights the potential value of this novel approach in enhancing surgical outcomes and improving patient safety. The findings demonstrate that the irrigation mist and CO_2_ system provides significantly superior intraoperative visualization, as reported by surgeons. The combination of CO_2_ and a fine irrigation mist helps maintain a clear surgical field and preserve tissue hydration, in contrast to the direct CO_2_ blower, which is associated with tissue desiccation and fragility. This improved visualization may also contribute to enhanced procedural efficiency, as indicated by the trend toward shorter operative times in the irrigation mist group. Although the difference in surgical times was not statistically significant, this may reflect the limited sample size rather than a lack of clinical impact. Improved visualization reduces the need for repeated field clearing, thus facilitating smoother anastomosis construction and procedural flow [6,7,8].

A significant finding of the study is the reduced incidence of ventricular fibrillation during aortic de-clamping in the irrigation mist group (10% vs. 40%). This benefit is likely due to the better preservation of myocardial stability and reduced stress on cardiac tissues, which can otherwise be exacerbated by desiccation and irritation caused by traditional CO_2_ blowers. Furthermore, the reduction in fibrillation may be linked to the system’s ability to lower micro-embolic gas activity, a known risk factor for myocardial and neurological complications [6,7]. Another notable result is the significant reduction in micro-embolic gas activity in the irrigation mist and CO_2_ group, as detected by transesophageal echocardiography (TEE). By minimizing gas bubble formation, the system addresses one of the most insidious risks of cardiac surgery—the potential migration of gaseous emboli to the coronary or cerebral circulation. This finding underscores the potential for the irrigation mist system to reduce postoperative neurological deficits and myocardial injury, although the absence of long-term follow-up data in this study limits a full assessment of these benefits. Future studies should include extended follow-up to evaluate the impact on cognitive and neurological outcomes comprehensively [7,8].

While the results underscore the promise of the irrigation mist and CO_2_ system, the study has limitations. As a retrospective analysis, it is prone to selection bias, which may have influenced the findings. Variations in patient characteristics or surgical technique, even if not statistically significant, could play a role. Additionally, the small sample size of 40 patients limits the statistical power, reducing the ability to generalize the results or detect subtler differences between groups. The subjective assessment of visualization quality by surgeons introduces potential observer bias, and future research should incorporate objective, quantitative metrics, such as automated imaging analysis, to validate this outcome [9,10]. The single-center nature of the study further limits the generalizability of its findings, as surgical outcomes may vary based on institutional protocols and surgeon experience. To address these issues, we plan to conduct a larger, multicenter, and prospective randomized controlled trial with a focus on eliminating selection bias, increasing sample size, and incorporating long-term follow-up data to better understand the clinical relevance of reduced micro-embolic activity [9]. Despite these limitations, the study provides compelling preliminary evidence supporting the use of the irrigation mist and CO_2_ system. Beyond its application in on-pump CABG, the system may offer benefits in other cardiac procedures, such as off-pump surgeries, where visualization and tissue preservation are critical. Expanding research to these areas could further validate the versatility and utility of this approach [10]. Finally, it is important to note that while this study evaluates a device (ClearView™ Blower/Mister), there are no conflicts of interest. The research was conducted without external funding, and no affiliations or financial relationships with the device manufacturer exist, ensuring the integrity and impartiality of the findings.

## 5. Conclusions

This retrospective study highlights the irrigation mist and CO_2_ system’s advantages over the traditional direct CO_2_ blower in on-pump coronary artery bypass grafting (CABG). In our experience, this brief report shows that the system significantly improved intraoperative visualization, reduced tissue desiccation, and was associated with a lower incidence of fibrillation during aortic de-clamping. Additionally, the irrigation mist and CO_2_ system demonstrated a notable reduction in micro-embolic gas activity, as detected by transesophageal echocardiography (TEE), suggesting that it may reduce the risk of neurological and myocardial complications related to gas embolization. These findings suggest that the irrigation mist and CO_2_, initially designed for off-pump coronary surgery, is also highly beneficial in on-pump settings, future research should focus on conducting a prospective, multicenter, and randomized controlled trial with a larger sample size and long-term follow-up to validate the findings of this preliminary analysis.

## Figures and Tables

**Table 1:** Patient characteristics.

Characteristic	Group 1 (Direct CO_2_ Blower)	Group 2 (Irrigation Mist and CO_2_)	*p*-Value
Number of Patients	20	20	-
Age (years)	65 ± 8	66 ± 9	0.65
Male Gender (%)	80%	85%	0.72
Body Weight (kg)	78 ± 10	77 ± 9	0.82
Height (cm)	170 ± 8	169 ± 9	0.78
Body Surface Area (BSA) (m^2^)	1.90 ± 0.15	1.88 ± 0.16	0.75
Diabetes (%)	40%	35%	0.78
Hypertension (%)	60%	65%	0.85
Hyperlipidemia (%)	55%	50%	0.80
Left Ventricular Ejection Fraction (LVEF %)	55% ± 7%	56% ± 6%	0.68

Note: values are presented as the n, mean, or percentage ± standard deviation (SD).

**Table 2:** Perioperative results.

Perioperative and Postoperative Outcomes	Group 1 (Direct CO_2_ Blower)	Group 2 (Irrigation Mist and CO_2_)	*p*-Value
Proximal Anastomoses (mean ± SD)	2.3 ± 0.6	2.4 ± 0.5	0.69
Distal Anastomoses (mean ± SD)	3.1 ± 0.8	3.2 ± 0.7	0.75
Y-Mammary Graft (%)	40%	50%	0.58
Sequential Graft (Mammary + Vein) (%)	60%	50%	0.65
Visualization Quality (Score)	3.8 ± 0.6	4.7 ± 0.5	<0.01
Incidence of Ventricular Fibrillation During Declamping (%)	8 patients (40%)	2 patients (10%)	0.03
Micro-Embolic Gas Activity (%)	10 patients (50%)	3 patients (15%)	0.02
Cardiopulmonary Bypass (CPB) Time (min)	85	77	0.05
Cross-Clamp Time (min)	67	65	0.72
Troponin Levels at 24 Hours (ng/L)	0.12 ± 0.03	0.11 ± 0.04	>0.05
Major Postoperative Complications (%)	None	None	-
Length of Postoperative Hospital Stay (days)	7.5 ± 1.2	7.0 ± 1.1	0.32

Note: values are presented as the n, mean, or percentage ± standard deviation (SD).

## Data Availability

The data presented in this study are available on request from the corresponding author.
